# Individual, social, and environmental influences on the transitions in physical activity among emerging adults

**DOI:** 10.1186/s12889-016-3368-3

**Published:** 2016-08-02

**Authors:** Kaigang Li, Danping Liu, Denise Haynie, Benjamin Gee, Ashok Chaurasia, Dong-Chul Seo, Ronald J. Iannotti, Bruce G Simons-Morton

**Affiliations:** 1 Department of Health & Exercise Science, Colorado State University, B 215E Moby Complex, Fort Collins, CO 80523 USA; 2 Division of Intramural Population Health Research, Eunice Kennedy Shriver National Institute of Child Health & Human Development, 6100 Executive Blvd, Bethesda, MD 20892-7510 USA; 3 University of Waterloo, 200 University Ave W, Waterloo, ON N2L 3G1 Canada; 4 Indiana University School of Public Health, Bloomington, IN 47405 USA; 5 The CDM Group, 7500 Old Georgetown Road, Bethesda, MD 20814 USA

**Keywords:** Moderate and vigorous physical activity, Emerging adults, Transition model, Physical activity planning, Peer physical activity

## Abstract

**Background:**

Youth’s physical activity (PA) may change across developmental periods. Although previous studies have observed a decline in levels of PA during adolescence, few studies have explored trends in PA during the transition from adolescence to young adulthood and what factors may impact the transitional change. The purpose of this study was to examine changes and predictors of change over time in PA from 10^th^ grade to post-high school.

**Methods:**

The NEXT Generation Health Study recruited a nationally-representative cohort of US 10^th^-graders, and administered longitudinal surveys in four waves (years) to follow up the participants to their first year after high school. Using transition models, the self-reported outcomes, moderate-to-vigorous PA (MVPA) and vigorous PA (VPA) each of which was repeatedly measured by one question, were modelled in association with wave-4 environmental-status variables and time-varying covariates.

**Results:**

There was a continuous decline in the proportion of respondents who met or exceeded the minimum recommended level for either MVPA (from 55.97 to 34.33 %) or VPA (from 65.96 to 54.90 %) from W1 to W4. Higher scores of peer PA, family support and VPA planning were prospectively associated with higher likelihood of meeting the MVPA/VPA recommendations. At wave 4, compared to those not working, attending 4-year colleges, or living on campus, participants working full/part time, not attending school or attending community-college level schools, and living at home or in own place were more likely to engage in MVPA.

**Conclusions:**

Peer PA, family support, self-regulatory skills, and environmental status after high school are critical factors that can promote MVPA/VPA among adolescents and emerging adults.

## Background

Physical activity (PA) is essential for the promotion of general health and the prevention of chronic health conditions in all ages, including adolescents and young adults [1]. Physical inactivity is well documented as a determinant of cardiovascular and metabolic health [2], type 2 diabetes, several forms of cancer, [3, 4] as well as the rising obesity epidemic among youth in the US [5]. The 2008 Physical Activity Guidelines (Guidelines) for Youth recommend that children and adolescents (17 years and younger) engage in 60 min of daily PA, most of which should be either moderate (M) or vigorous (V), aerobic PA and should include VPA at least 3 days a week [1]. The Guidelines for Adults recommend that adults (18 years and older) should engage in at least 150 min a week of MVA (alternatively 75 min/week of VPA) for health benefits and/or at least 300 min a week of MVA (alternatively 150 min/week of VPA) for additional and more extensive health benefits. However, less than 20 % of U.S. adolescents (17 years and younger) meet the recommended level of aerobic PA [6]. For mix-age US youth (9^th^ to 12^th^ grade) less than 30 % engaged in MPA at least 60 min/day on all seven days per week and less than 50 % on five or more days per week [7]. The developmental period from adolescence to young adulthood is characterized by a surprisingly high risk of obesity (prevalence of obesity = 22.1 % with body mass index ≥ 30) [8]. Moreover, it is a critical period marked by sharp declines in PA [9–11]. However, to our knowledge, no studies have investigated the transitional change of youth PA engagement and potential determinants of the change at each stage controlling for the behavior and other covariates in the prior stage (i.e., the PA engagement and other covariates in the previous wave).

Some evidence suggests that certain psychosocial and social-contextual variables, such as peer influence, family support, and action planning [12–14] are positively associated with levels of PA in high school. Only a few studies have examined longitudinal changes [15] and trajectories [11] of PA and their temporal and prospective predictors with limited causal conclusions, but no transitional associations were tested. In addition, no studies have investigated differences according to environmental status after high school.

Generally, a transition model estimates the probability of a categorical response (e.g., meeting PA recommendations) given the past responses, and explores the covariates’ effect on the transition probability [16]. More specifically, it estimates the average of most proximal past measurement of covariates on the outcome of interest (e. g., PA at time 1 on PA at time 2, time 2 on time 3, …, time n on time n + 1), and captures the factors affecting behavioral change over time.

Overall, the objective of this study is to identify the determinants of PA during the transition from adolescence to adulthood among emerging adults, specifically the year after high school. The specific aims were to examine across the four annual waves from 10th grade to one year after high school: (1) changes in self-reported PA (including MPA to VPA [MVPA] and VPA); and (2) predictors of these changes, including perceived peer PA, family support for PA, VPA planning, and post-high school environmental status (school status, work status, and residence).

## Methods

### Sampling

This longitudinal analysis examines data from Wave 1 (W1, 10^th^ grade) through W4 (1^st^ year after high school) of the NEXT Generation Health Study, a nationally-representative longitudinal US study starting in the 2009–10 school year. The participants in this study are termed as emerging adults because they transition from adolescence (W1-W3, i.e., grades 10 to 12) to early adulthood (W4; year after high school). Primary-sampling units were stratified by the nine US census divisions. Within each census division, the sample of primary sampling units was first selected with probability proportional to the total enrollment. Within this sampling framework, 145 schools with 10th grade were randomly recruited and 81 (55.9 %) agreed to participate. A total of 2785 participants completed the yearly survey in all four waves, with response rates of 91 % (not 100 % at W1 because 260 more participants were recruited from W2), 88, 86 and 78 % at W1 to W4, respectively. Parental consent or participant’s assent was obtained; participant consent was obtained upon turning 18. African American participants were oversampled to increase the accuracy of the analysis for this population. The study protocol was approved by the Institutional Review Board of the *Eunice Kennedy Shriver* National Institute of Child Health and Human Development.

### Measures

*Physical activity* was measured by two survey questions. To measure *MVPA*, we asked participants how often they were physically active for a total of at least 60 min per day over the past 7 days (response options ranged from 0 to 7 days). Before participants recalled their MVPA, a statement was highlighted to remind them what activities they should think about, namely, “Physical activity can be done in sports, school activities, playing with friends, or walking to work or school. Some examples of physical activity are running, brisk walking, rollerblading, biking, dancing, skateboarding, swimming, soccer, basketball, football, & surfing. For this next question, add up all the time you spent in physical activity each day.” The question was from the Youth Risk Behavior Surveillance (YRBS) survey [16]. The MVPA scores were dichotomized to reflect those who engaged in at least 60 min per day on 5 or more days (indicated as meeting MVPA recommendation hereafter) vs. those who did not. We set this cutoff point because the 2008 Guidelines [1] recommend that “adults should increase their aerobic physical activity to 300 min a week of moderate-intensity” and YRBS reported prevalence of youth graded 9–12 using the 60 min/day on at least 5 days a week as one of cut points [7]. Given that most the participants in the study turned 18 years old after W2, the use of the cutoff point of “60 min/day on at least 5 days a week” made it possible to compare MVPA engagement within the NEXT cohort longitudinally from adolescence to early adulthood in the same cohort and compare NEXT data with the national data from YRBS cross-sectionally [7].

A separate question asked participants how many hours a week they typically engaged in *VPA*. A highlighted statement reminds participants of what activities they should recall for VPA, namely, “Vigorous physical activity is any activity that increases your heart rate and makes you get out of breath some of the time. For this next question, add up all the time you spent in vigorous physical activity each day.” The response options include none, about half hour, about 1 h, about 2 to 3 h, about 4 to 6 h, and 7 h or more. The validity of this question from the Health Behavior in School-Aged Children survey was established previously [17]. The VPA scores were dichotomized to indicate those who engaged in vigorous PA for at least 2 h (using option “about 2 to 3 h” in the questionnaire) a week (indicated as meeting VPA recommendation hereafter) vs. those who did not. The use of this criterion approximately reflects the 2008 Guidelines that adults (18 years and older) should do 150 min (two and half hours) a week of VPA for more extensive health benefits [1]. Therefore, this criterion made it possible to compare the VPA engagement within the NEXT cohort longitudinally given that the participant age spanned the period from adolescence to adulthood.

*VPA planning* was measured using three previously-validated items [18]. Participants were asked how often in the past seven days they planned for VPA, which included when, how often (i.e., the frequency), and where they planned to exercise (from 1 = not at all to 5 = very often). The mean score of the three items was calculated at each wave. For the current sample, Cronbach alpha internal consistency coefficients of this scale were 0.90, 0.93, 0.94, and 0.94 for W1 to W4, respectively.

*Peer physical activity* (W1 to W4) was measured with one item, which was derived from the National Longitudinal Study of Adolescent Health [19] and revised for this study. We asked participants how often their five closest friends engaged in VPA at least 3 times a week (from 1 = never to 5 = almost always).

One item was used to measure *student-perceived parental support of daily PA* in W1 through W3. The question was derived from the National Survey on Drug Use and Health [20] and asked participants how important it was to their parents/guardians that he or she get daily PA and/or exercise (from 1 = not at all to 7 extremely).

Self-reported weight (in kilograms) and height (in meters) were used to calculate *Body mass index (BMI)* (kg/m^2^).

Three *environmental status variables* (three categories each) *at W4* were assessed: *residence*, *school status*, and *work status*. Residence included parent/guardian’s home, own place (rented room, apartment), and on campus (school dormitory or residence hall, fraternity/sorority house). School status consisted of not in school, technical/community college, and university or college. Work status included not working, part time (<30 h), and full time (≥30 h).

The *demographic variables* included sex, race/ethnicity, family socioeconomic status, urbanicity, and parent education. Family socioeconomic status was estimated using the Family Affluence Scale [21] and participants were categorized as low, moderate or high affluence [22]. Participants’ schools were ranked in the baseline wave of the study according to a seven-level scale ranging from large central city to rural area. Those attending schools in a rural area were categorized as rural, and the remaining categories were classified as urban. Parent education reflecting the highest of up to two parents’ educations was reported by the parent completing the consent form (< high school diploma, high school diploma/GED, some college/technical school/advanced degree, or a bachelors/graduate degree).

### Statistical analysis

Overall, using four waves of data (W1 to W4) from NEXT our investigation employed transition models [16] to track the development of PA over time by accounting for the autocorrelation of repeated measures of covariates and outcomes. Of the total sample of 2785 participants, 126 participants who were still in high school at W4 or self-reported other residences were excluded from this analysis because each group had too few to analyze and these environments represented qualitatively different life circumstances. The dichotomized MVPA and VPA variables represent the two outcomes of interest.

Multiple imputation by chained equations based on the assumption of missing at random [23, 24] was used to impute missing outcome and independent variables. The algorithm recursively imputed each missing variable by estimating its distribution conditional on other variables. A total of 50 imputed data sets were generated using IVEware software package [25].

In an imputed data set, each subject contributes three transitions in consecutive years, from W1 to W2, W2 to W3, and W3 to W4. Then the probability of a response in one wave (e.g., MVPA at W4) is written as a regression function of the response at the previous wave (e.g., MVPA at W3), and other risk factors at previous wave (e.g., VPA planning at W3). For model simplicity, we made a “Markov assumption” that the state in a particular wave is dependent on the state in the most recent previous wave and not the more distant ones so only one previous wave was included in the regression functions [26]. This assumption is commonly used in transition models, particularly in studying life history events in social sciences [27, 28]. In our study, the transition model is analogous to three logistic regression models for three transitions, but we estimate these logistic regressions jointly through generalized estimating equations (GEE), with the assumption that the effect of the covariates remains constant over time. GEE is a commonly used approach to estimate the binary transition model [29, 30]. Relatively few model assumptions are needed: as long as the Markov assumption holds, the population-average transition probability can be estimated consistently. Robust variance estimator was calculated to account for multiple transitions from the same subject [31]. The coefficients of the transition models are interpreted as the population-average effect of a covariate at the previous wave on the PA outcomes at the current wave, while other variables in the previous wave held fixed. The estimation of regression coefficients was implemented in SAS PROC GENMOD, with MVPA and VPA examined in separate models for all regressions. Features of complex survey design including clustering and sampling weights were taken into account to make these results representative and comparable to other nationally representative surveys. More details about transitional models are shown in Appendix.

Complete imputed data (N = 2659) were analyzed in four steps: (1) descriptive analyses were performed to examine the percentage of participants meeting MVPA and VPA criteria in each of the four waves and changes across waves; (2) bivariate logistic regressions were used to examine associations of PA outcomes with demographic variables at each wave; more traditional levels such as *p* = 0.05 can fail in identifying potentially important covariates, therefore a larger *p* value, i.e., *p* = .25, was used as an inclusion criterion (association at *p* = .25 level in at least one wave) for subsequent multivariate models [32]; (3) multivariate logistic models were estimated separately for W2, 3, and 4 with PA outcomes regressed on previous wave PA and covariates controlling for selected demographic variables in step 2; (4) multivariate transition models were estimated with the PA outcomes regressed on previous wave PA and covariates controlling for demographic variables (Model 1 in Tables 2 and 3); and (5) environmental status variables were added to the models in step 4 to test their association with the outcomes at W4, by including interaction terms (without main effects) between W4 and each of environmental status variables in separate models (Models 2–4 in Tables 2 and 3). The interaction term is interpreted as the impact of environmental variables on the PA outcomes at W4. The analysis was repeated for each of the 50 imputed data sets. Then the results were combined using Rubin’s combination rule [23], implemented in SAS PROC MIANALYZE.

## Results

### Descriptive analysis

At W1, the 2659 participants had a weighted mean age of 16.20 years (SE = 0.02). Descriptive information of demographic (W1) and environmental (W4) variables is presented in Table 1 (weighted results based on the imputed data were reported and the same hereafter).

**Table 1:** Descriptive information of demographic (wave 1) and environmental (wave 4) variables (N = 2659)

	Wave 1		Wave 4		Met recommended MVPA at Wave 4^a^		Met recommended VPA at Wave 4^a^	
	Weighted %	SE	Weighted %	SE	Weighted %	SE	Weighted %	SE
Wave								
Wave 1	–	–	–	–	55.79	1.68	65.74	2.07
Wave 2	–	–	–	–	48.06	2.10	59.43	2.22
Wave 3	–	–	–	–	43.50	1.97	56.67	2.03
Wave 4	–	–	–	–	33.99	1.86	54.41	2.26
Sex								
Female	55.04	1.58	–	–	–	–	–	–
Male	44.96	1.58	–	–	–	–	–	–
Race/ethnicity								
White	56.46	5.96	–	–	–	–	–	–
Hispanic	18.50	3.67	–	–	–	–	–	–
Black	20.25	4.48	–	–	–	–	–	–
Other	4.80	0.90	–	–	–	–	–	–
Family affluence								
Low	23.38	2.83	–	–	–	–	–	–
Moderate	49.01	1.67	–	–	–	–	–	–
High	27.61	2.63	–	–	–	–	–	–
Parent, highest education								
<high school diploma	8.22	2.11	–	–	–	–	–	–
High school diploma or GED	24.60	2.11	–	–	–	–	–	–
Some degree	39.44	1.67	–	–	–	–	–	–
Bachelor’s or graduate degree	27.74	2.90	–	–	–	–	–	–
Rural/urban at wave 1								
Urban	65.80	7.50	–	–	–	–	–	–
Rural	34.20	7.50	–	–	–	–	–	–
Work status								
Not working	–	–	50.87	2.95	27.70	2.47	57.76	2.59
Work part time <30 hours	–	–	34.40	1.93	36.81	3.23	53.84	3.89
Work full time ≥30 hours	–	–	14.73	2.04	49.22	4.17	61.51	5.22
School status								
College/Graduate School	–	–	49.00	8.14	29.59	2.53	56.40	3.18
Not attending school	–	–	24.63	4.03	40.50	40.9	51.49	4.08
Tech/Voca/Comm	–	–	26.37	4.63	36.06	3.21	53.41	3.17
Residence								
On campus	–	–	34.11	12.27	30.36	3.35	60.84	4.26
At home	–	–	47.89	10.57	35.17	2.51	51.28	2.70
In own place	–	–	18.00	10.45	38.00	4.95	51.17	6.42

*Tech/Voca/Comm* technological or vocational school or community college, *MVPA* moderate to vigorous physical activity, *VPA* vigorous physical activity

^a^The declines between adjacent waves were significant (*p* < .05 to .001) for both MVPA and VPA, with the exceptions of waves 2 to 3 (*p* = .07) and waves 3 to 4 declines (*p* = .29) for VPA

The weighted percentages of participants who engaged in MVPA (for 60 min/day on at least 5 days a week) and VPA (for at least 2 h a week) declined from W1 through W4 continuously (Table 1). Percentage of participants who engaged in MVPA for 60 min/day on at least 5 days a week and VPA for at least 2 h a week at W4 by environmental variables are shown in Table 1.

### PA transition by wave

**Fig. 1:**
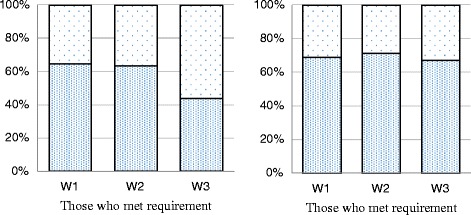
MVPA and VPA Transitions: among participants who MET the requirements, percentage who met and did not meet the requirements the next year. **a** MVPA. **b** VPA

**Fig. 2:**
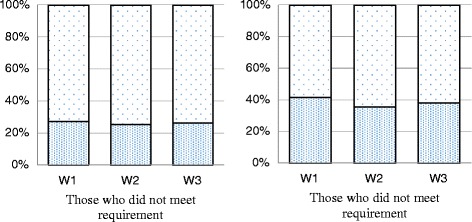
MVPA and VPA Transitions: among participants who DID NOT meet the requirements, percentage who met and did not meet the requirements the next year. **a** MVPA. **b** VPA

Figures 1 and 2 illustrate stability of MVPA and VPA across waves. The proportion of participants who engaged in recommended MVPA (Fig. 1a) was relatively maintained high and stable from W1 to W2 (65 %) and from W2 to W3 (65 %) but decreased from W3 to W4 (44 %). Proportion of participants engaged in recommended VPA maintained stable across all waves, with 65 to 67 % of those meeting the criteria at a particular wave also meeting the criteria in a subsequent wave (Fig. 1b). Among those who did not meet the criteria of MVPA at a given wave, 24–27 % met the criteria in subsequent waves (Fig. 2a). For VPA, the percentage of those who transitioned from not meeting to meeting the criteria across waves ranged from 35 to 41 % (Fig. 2b).

### Bivariate models by wave

In bivariate analyses all five demographic variables were associated at *p* ≤ .25 level with both MVPA and VPA in at least one wave of data (data not shown) so were included in subsequent multivariate models.

### Transition models

Transition models (Tables 2 and 3) were estimated with MVPA and VPA regressed on explanatory variables and covariates controlling for demographic variables (Model 1) and in turn with addition of each environmental status variable in separate models (Models 2, 3, 4).

**Table 2:** Transition models of moderate-to-vigorous physical activity

	Model 1^a^			Model 2^a^			Model 3^a^			Model 4^a^		
	AOR	95 % CI		AOR	95 % CI		AOR	95 % CI		AOR	95 % CI	
Wave												
2	Ref			Ref			Ref			Ref		
3	0.95	0.80	1.12	0.91	0.70	1.20	1.00	0.77	1.30	1.00	0.74	1.34
4	0.63^***^	0.51	0.76	0.46^***^	0.34	0.63	0.43^***^	0.30	0.61	0.39^***^	0.26	0.59
Variables in previous wave												
Five closest friends PA	1.11^**^	1.03	1.19	1.11^**^	1.03	1.19	1.11^***^	1.04	1.19	1.11^**^	1.03	1.19
VPA planning	1.14^***^	1.06	1.22	1.14^***^	1.06	1.23	1.15^***^	1.07	1.24	1.15^***^	1.07	1.23
Body mass index	0.99	0.98	1.01	0.99	0.98	1.01	0.99	0.97	1.01	0.99	0.98	1.01
Parent support to PA	1.09^***^	1.03	1.14	1.09^***^	1.03	1.15	1.09^***^	1.03	1.15	1.08^***^	1.03	1.14
MVPA 60 min/day 5days per week												
No	Ref			Ref			Ref			Ref		
Yes	2.83^***^	2.34	3.41	2.84^***^	2.35	3.44	2.85^***^	2.36	3.45	2.85^***^	2.35	3.45
Interaction of wave 4 × environmental status												
Work status												
W4 × Not working	–	–	–	Ref			–	–	–	–	–	–
W4 × Work part time <30 hours	–	–	–	1.52^*^	1.00	2.30	–	–	–	–	–	–
W4 × Work full time ≥30 hours	–	–	–	2.47^***^	1.60	3.81	–	–	–	–	–	–
School status												
W4 × College/Graduate School	–	–	–	–	–	–	Ref			–	–	–
W4 × Not attending school	–	–	–	–	–	–	2.18^***^	1.35	3.50	–	–	–
W4 × Tech/Vocational School/Community College	–	–	–	–	–	–	1.79^***^	1.21	2.63	–	–	–
Residence at W4												
W4 × On campus	–	–	–	–	–	–	–	–	–	Ref		
W4 × At home	–	–	–	–	–	–	–	–	–	1.65^*^	1.00	2.73
W4 × In own place	–	–	–	–	–	–	–	–	–	1.73^*^	1.10	2.71

*AOR* adjusted odds ratio, *CI* confidence interval, *MVPA* moderate to vigorous physical activity, *VPA* vigorous physical activity

^*^ *p* ≤ .05, ^**^ *p* ≤ .01, ^***^ *p* ≤ .001, ^a^the model was controlled for demographic variables and included variables listed

**Table 3:** Transition models of vigorous physical activity

	Model 1^a^			Model 2^a^			Model 3^a^			Model 4^a^		
	AOR	95 % CI		AOR	95 % CI		AOR	95 % CI		AOR	95 % CI	
Wave												
2	Ref			Ref			Ref			Ref		
3	1.04	0.88	1.23	0.93	0.70	1.23	1.05	0.77	1.42	0.86	0.60	1.25
4	0.96	0.78	1.18	0.97	0.74	1.26	0.80	0.60	1.07	1.04	0.73	1.48
Variables in previous wave												
Five closest friends PA	1.17^***^	1.09	1.27	1.18^***^	1.09	1.27	1.17^***^	1.08	1.27	1.17^***^	1.08	1.26
VPA planning	1.23^***^	1.14	1.32	1.22^***^	1.14	1.32	1.23^***^	1.14	1.33	1.22^***^	1.13	1.32
Body mass index	0.99	0.98	1.00	0.99^†^	0.97	1.00	0.99	0.98	1.00	0.99	0.98	1.00
Parent support to PA	1.08^**^	1.02	1.14	1.08^***^	1.03	1.14	1.07^***^	1.02	1.13	1.08^**^	1.02	1.13
VPA ≥ 2 hours a week												
No	Ref			Ref			Ref			Ref		
Yes	2.43^***^	1.81	3.27	2.44^***^	1.82	3.27	2.42^***^	1.80	3.25	2.40^***^	1.79	3.22
Interaction of wave 4 × environmental status												
Work status												
W4 × Not working	–	–	–	Ref			–	–	–	–	–	–
W4 × Work part time <30 hours	–	–	–	0.92	0.64	1.32	–	–	–	–	–	–
W4 × Work full time ≥30 hours	–	–	–	1.29	0.78	2.14	–	–	–	–	–	–
School status												
W4 × College/Graduate School	–	–	–	–	–	–	Ref			–	–	–
W4 × Not attending school	–	–	–	–	–	–	1.11	0.75	1.65	–	–	–
W4 × Tech/Vocational School/Community College	–	–	–	–	–	–	1.13	0.75	1.70	–	–	–
Residence at W4												
W4 × On campus	–	–	–	–	–	–	–	–	–	Ref		
W4 × At home	–	–	–	–	–	–	–	–	–	0.80	0.43	1.47
W4 × In own place	–	–	–	–	–	–	–	–	–	0.89	0.61	1.30

*AOR* adjusted odds ratio, *CI* confidence interval, *VPA* vigorous physical activity

^*^ *p* ≤ .05, ^**^ *p* ≤ .01, ^***^ *p* ≤ .001,^†^<.05, *p* <.10 ^a^the model was controlled for demographic variables and included variables listed

Transition models for MVPA are shown in Table 2. Model 1 examined prospective associations of the previous wave covariates, excluding the environmental variables. For Models 2-4, environment variables have been added to Model 1, sequentially, when the response of interest is at W4. This was done by including interaction of indicator for W4 with the environment variable. In Model 1, the likelihood of meeting the MVPA recommendation was significantly higher among those with greater peer PA (AOR = 1.11, *p* < .01), VPA planning (AOR = 1.14, *p* < .001), parental support of PA (AOR = 1.09, *p* < .001), and who met the MVPA recommendation in the previous wave (AOR = 2.83, *p* < .001). In all three models including the environmental variables, peer PA and parental support of PA, VPA planning, and MVPA in the previous wave, were still significantly associated with MVPA. In Model 2, participants working part time (AOR = 1.52, *p* < .05) and full time (AOR = 2.47, *p* < .001) at W4 were more likely to meet the MVPA recommendation compared to those not working at W4. In Model 3, participants not attending schools (AOR = 2.18, *p* < .001) and attending technical or community college (AOR = 1.79, *p* < .001) compared to those who attended college at W4 were more likely to meet the MVPA recommendation at W4. In Model 4, participants living at home (AOR = 1.65, *p* < .05) and living in own place (AOR = 1.73, *p* < .05) compared to those living on campus were more likely to meet the MVPA recommendation at W4.

Transition models for VPA are shown in Table 3. Model 1 examined predictive effects of the previous wave covariates on VPA in the current wave, controlling for demographic variables; Models 2–4 retained the variables from Model 1 and added each of the environmental variables. In Model 1, Peer PA (AOR = 1.17, *p* < .001) and parental support of PA (AOR = 1.08, *p* < .01), VPA planning (AOR = 1.23, *p* < .001), and VPA in the previous wave (AOR = 2.43, *p* < .001) were significantly associated with meeting the VPA recommendation. In Models 2, 3 and 4, none of the interaction terms were significant, indicating no association of the environmental status variables with VPA.

## Discussion

This study used a nationally representative sample to examine the longitudinal change of MVPA and VPA among youth during their transition from adolescence into early adulthood. To our knowledge, this is the first-time application of a transition model approach to test how change in PA can be explained by psychosocial, social and environmental variables. We found that engagement in MVPA, but not VPA, continuously declined from 10^th^ grade to the first year post-high school, which is consistent with previous findings [11]. Similar to previous findings for adolescents [12–14], psychosocial variables including perceived parent support of PA, perceived peer PA engagement, and VPA planning strongly, were associated with increased likelihood of engaging in MVPA and VPA. Findings also indicate that environmental variables were associated with levels of MVPA one year after high school. Specifically, those who were working, were not attending college, and were not living on campus, were more likely to engage in MVPA at W4.

While previous studies have observed a decline in levels of PA during adolescence, few studies have explored trends in PA during the transition from adolescence to young adulthood [9, 15]. Our findings are consistent with two Canadian cohort studies [9, 11], which similarly observed declines in PA during this transition. Given that MVPA declined in both high-school and post high-school contexts, more research is needed to explore the mechanism resulting in, as well as protecting against, the steady declined MVPA during the transition.

Consistent with a previous international cohort study [33], our findings confirm the importance of encouraging the maintenance of consistent levels of PA throughout this developmental period. The joint findings display the need for establishing and promoting sustainable strategies to initiate and maintain youth PA engagement over the developmental period.

A number of cross-sectional studies have documented that parent [34, 35] and peer [36] social influences are positively associated with increased levels of PA. A limited number of longitudinal data also demonstrate that parental support [37] and peer/friend PA behavior [4] positively influence PA engagement among adolescents. However, there is a paucity of longitudinal evidence during the transition period from high school to the first year after high school. A review study [38] examined parent influence on drinking of the first-year college students and found that high parental monitoring and disapproval of alcohol use were negatively associated and parental permissiveness was positively associated with alcohol use. In addition, parentally imposed stringent drinking limits attenuated the powerful facilitating effects of peers on drinking. The current study extended the findings from alcohol drinking to a healthful behavior, i.e., PA, and upheld the continued parent and peer influence on emerging adults. We conducted additional analyses to test the potential moderation effect (including interaction term of parent support × peer PA in the model) of parent support and peer influence on PA, however the data did not show a significant moderation effect. Given the methodological strengths of the current study, our findings provide compelling evidence of the continued importance of parent and peer support in levels of PA.

A body of evidence suggests that action planning (the act of consciously scheduling and/or arranging to engage in a behavior) provides a bridge between intention to engage in PA and actual engagement with PA [18, 39]. A recent cross-sectional study found that those who planned for PA were more likely to engage in PA [14]. Moreover, we also observed prospective associations between planning and both MVPA and VPA, in the current study. These findings are consistent with previous studies suggesting the importance of planning [40]. Given the demands, stresses and increased levels of independence associated with beginning college, action planning may be an important strategy, particularly important for first-year college students, to implement.

In addition to psychological and social determinants, environmental context may provide variable opportunities and barriers to youth engagement in PA [41]. There are a limited number of studies that have explored the extent to which a recent change in environmental status, such as residence, school status, and work status, influence levels of PA during the transition from adolescence to early adulthood. A cross-sectional study among Australian college students examining the association between environmental status and levels of PA found that females who worked were more likely to engage in sufficient levels of PA compared to female students who did not work [42]. The findings from our longitudinal study, consistent with previous findings, document the importance of environment and raise intriguing questions about its role.

Curiously, in the transition models, student part-time and full-time work status at W4, relative to not working, were significantly associated with MVPA. Yet, the mechanism for employment being related to more engagement of MVPA is still unclear. In the Australian study, the authors proposed that a job commitment may lead to better organization and time management, increasing the likelihood of PA participation [42]. Alternatively, there may be something unique about the motivation of youth who work that also contributes to motivation to engage in PA. However, our data did not support that hypothesis, at least regarding PA planning. We conducted an additional analysis to examine the interaction of VPA planning with work status in the MVPA transition model and the association between VPA planning and work status in W4 data, but no significant results were observed in either test (data not shown). The relationship between work status and MVPA was not replicated in models assessing VPA. The item used to capture MVPA did not specify the activity type, i.e., organized PA, recreational PA or occupational PA; it is plausible that occupational PA may play a role in increasing levels of MVPA in this population of emerging adults. That is, the types of jobs likely to be obtained among this age group, including service sector and jobs requiring physical labor, contribute to the amount of physical activity acquired. However, future studies are needed to confirm the current findings and further explicate the relationship between work and PA.

Our findings indicate that those attending traditional 4-year colleges were less likely to engage in MVPA than those not attending colleges, technical schools, or community colleges. No significant associations were found between school status and VPA. Given the similar pattern with the association of work status with MVPA and VPA, we posit that the association between school status and MVPA may be work-related, in that more participants not attending school reported working full or part time (64.7 % of participants not attending college, 53.8 % attending technical/community school, and 38.8 % attending college). Additional research investigating why those attending 4-year colleges are less likely to engage in adequate MVPA is warranted.

Previous studies suggest that access and quality of PA resources may influence engagement with MVPA and VPA in the general population [43]. Many college campuses in the US enable MVPA and VPA through structure and design (spacious campuses conducive walking and bicycling), facilities (recreational centers, outdoor and indoor courts), and extracurricular resources (intramural sports and fitness clubs) [44]. Notably, studies have found that accessibility and proximity of exercise facilities were positively associated with students’ PA engagement [45, 46], but PA was higher among those at on-campus and off-campus settings [45]. According to the results of the current study, residence may have different effects on MVPA and VPA. In the multivariate logistic regression model living on campus is positively associated with VPA and not associated with MVPA. In the transition models, the association of campus residence with MVPA was significant, and the association with VPA was not. It is possible that participants who lived on-campus during their first-year of college take advantage of campus-related amenities for PA; however the residence effect may be suppressed by their individual determinants such as social support and planning or others (e.g., academic and other school-related or competing commitments) not measured in this study. Those who lived off-campus during their first-year of college may take advantage of non-campus related amenities they had prior familiarity with, such as parks and local fitness centers, to engage in MVPA. Further research should explore more specific residence-related factors determining the engagement of MVPA and VPA for first-year high-school graduates.

There are several limitations of this study. First, our measures of MVPA did not differentiate between different types of activities (such as competitive or recreational exercise), and everyday activities (such as active transportation and job-based activities). Second, regardless of its wide use, the two self-reported questions derived from YRBS may not estimate the proportion of recommended moderate PA and vigorous PA accurately among youth, with one previous study reporting that study participants overestimate how vigorous their activities were compared with objectively measured PA) [47]. Third, the single-item measure of parental support on participant PA as well as peer physical activity may not capture all dimensions of the constructs. Fourth, we did not have a measure of access to PA facilities, which may have helped with understanding of the environmental variables.

The main strengths of this study include prospective longitudinal design encompassing the transition from adolescence to young adulthood, a nationally-representative sample which increases generalizability of our findings, and multiple social and environmental variables, providing a more comprehensive understanding of potential intervention targets.

### Implications for PA promotion among youth

A large body of literature has documented that 60 min or more of MVPA is developmentally appropriate and enjoyable to school-age youth [48], and school-based [49] and/or family and community combined [50] interventions can increase regular participation in PA among high-school students. However, few studies have examined the dynamic change in the transitional period from adolescence to early adulthood, and identified the intervention targets appropriate to this group specifically in this period of time. The main findings of this study are that MVPA engagement decreased from high school to one year after high school and was associated with previous MVPA engagement and social contextual factors during this transitional period. Based on the findings, health professionals and administrators in both high schools and post-high school organizations (e.g., universities, worksites) recognize the need for interventions that would foster sustained PA engagement. PA promotion in high schools may be particularly important to the extent that it may be easier to foster maintenance than initiation of PA. Comprehensive PA intervention programs including social level factors (i.e., schools/universities, parents, peers, and environments) and individual level factors (i.e., planning skills) are needed to promote and sustain youth PA more effectively.

In addition, the findings that the environmental variables were associated with MVPA only may suggest the measurement of MVPA may account for “incidental” unplanned PA compared to VPA. In other words, while planning was important for both PA types, environmental variables were significant only for MVPA, possibly because it is more sensitive to environmental influences during the transitional first year after high school. These findings also raised questions, “why did environment impact first-year college students’ VPA poorly despite generally great facilities and flexible schedules? Is this about not having time, competition from other activities, or some strange norm among college students?” The answers to those questions may help university health administrators and educators to design and implement tailored program to freshmen particularly.

## Conclusion

Our data indicate that peer PA engagement, family support, self-regulatory skills, and environmental status after high school are critical factors that can promote MVPA and/or VPA among adolescents and emerging adults. Action planning and social influences may be particularly important targets of intervention. Interventions that promote PA in high schools may be also particularly important to the extent that it may be easier to foster maintenance than initiation of PA.

## Abbreviations

MPA, moderate PA; MVPA, moderate-to-vigorous PA; PA, physical activity; VPA, vigorous PA

## Appendix. Details of the transition models

Let ${Y}_{ij}$ denote the binary outcome (i.e., moderate-to-vigorous physical activity [MVPA], vigorous physical activity [VPA]) for subject $i$ at wave $j$. Under Markov assumption, the transition model estimated the transition probability, $P\left({Y}_{ij}=1\Big|{Y}_{i,j-1}\right)$, and relates this probability to covariates. The lagged response, ${Y}_{i,j-1}$ was included in the model as a covariate. In our study, there were three transitions from consecutive waves with $j=2,3,4$, i.e., $P\left({Y}_{i2}=1\Big|{Y}_{i1}\right)$, $P\left({Y}_{i3}=1\Big|{Y}_{i2}\right)$, and $P\left({Y}_{i4}=1\Big|{Y}_{i3}\right)$. The transition model is given as below:

1 $$ P\left({Y}_{ij}=1\Big|{Y}_{i,j-1},{X}_{i4},{W}_{i,j-1},{Z}_i\right)={\beta}_{0j}+{\beta}_1{Y}_{i,j-1}+I\left(j=4\right)\times {X}_{i4}^{\mathit{\hbox{'}}}{\beta}_2+{W}_{i,j-1}^{\mathit{\hbox{'}}}{\beta}_3+{Z}_i^{\mathit{\hbox{'}}}{\beta}_4, $$

where ${Z}_i$ is the design vector of the baseline demographic covariates (i.e., sex, race/ethnicity, family affluence, and highest education of either parent), ${W}_{i,j-1}$ is the time varying factors in the previous wave (i.e., five closest friends PA, sedentary behavior, body mass index, parent support to PA, VPA planning in Wave $j-1$), ${X}_{i4}$ is the environmental variables at W4 (school, residential and work status). We first excluded the environmental variables in the transition model, referred to as Model 1; then each environmental variable was added in separately, referred to as Models 2, 3, and 4. The wave-specific intercept ${\beta}_{0j}$ is actually the main effect of wave, reflecting that the transition probabilities varied with time. The interaction term between wave = 4 and environmental variables is $I\left(j=4\right)\times {X}_{i4}^{\hbox{'}}$, meaning that this term only enters the model when the outcome is at W4. We could write out the three transition probabilities as follows:

2 $$ P\left({Y}_{i2}=1\Big|{Y}_{i1},{X}_{i4},{W}_{i1},{Z}_i\right)={\beta}_{02}+{\beta}_1{Y}_{i1}+{W}_{i1}^{\mathit{\hbox{'}}}{\beta}_3+{Z}_i^{\mathit{\hbox{'}}}{\beta}_4 $$

3 $$ P\left({Y}_{i3}=1\Big|{Y}_{i2},{X}_{i4},{W}_{i2},{Z}_i\right)={\beta}_{03}+{\beta}_1{Y}_{i2}+{W}_{i2}^{\mathit{\hbox{'}}}{\beta}_3+{Z}_i^{\mathit{\hbox{'}}}{\beta}_4 $$

4 $$ P\left({Y}_{i4}=1\Big|{Y}_{i3},{X}_{i4},{W}_{i3},{Z}_i\right)={\beta}_{04}+{\beta}_1{Y}_{i3}+{X}_{i4}^{\mathit{\hbox{'}}}{\beta}_2+{W}_{i3}^{\mathit{\hbox{'}}}{\beta}_3+{Z}_i^{\mathit{\hbox{'}}}{\beta}_4 $$

Note that ${X}_{i4}$ only predicts the transition from W3 to W4. Table 2 in the paper showed the results of *separately* estimating the models (2)-(4) in each wave. The transition models in Tables 3 and 4 *jointly* estimated (2)-(4) (hence fit the model (1)) using generalized estimating equations, accounting for the clustering of three transition probabilities from the same subject.
